# Non-volant small mammal data from fragmented forests in Terengganu State

**DOI:** 10.1016/j.dib.2018.10.061

**Published:** 2018-10-24

**Authors:** Nurul Khaleeda Abd Khalib, Nur Juliani Shafie, Hasrulzaman Hassan Basri, Bryan Raveen Nelson, Mohd Tajuddin Abdullah

**Affiliations:** a School of Marine and Environmental Sciences, Universiti Malaysia Terengganu, 21030 Kuala Nerus, Terengganu, Malaysia; b Institute of Tropical Biodiversity and Sustainable Development, Universiti Malaysia Terengganu, 21030 Kuala Nerus, Terengganu, Malaysia

**Keywords:** Herbivore, Cage trap, Tropics, Catch and release, Understory

## Abstract

This data article is about non-volant small mammal (squirrel, rat and tree shrew) capture from fragmented forest understories within sub-urban areas of Setiu (Peladang Agro Resort and Setiu Wetland Research Station) and inhabited areas of Hulu Terengganu (Saok and Lasir waterfalls) that are situated in Terengganu State, Peninsular Malaysia. Fruits like banana and oil palm were individually placed into each cage before the cages were fastened onto three to five meter height tree branches. The traps were also spatially distributed about ten meters from each other. Under this installation, fifty baited traps were used during the twenty-four nights of sample collection. All animals caught were distinguished by morphology and released at the same location it was caught. The understory data comprise of seven non-volant mammal species from family groups Sciuridae, Muridae and Tupaiidae. Overall, *Callosciurus notatus* (*n* = 17, 39%) were dominant in the capture pool from all sites. Comparatively, *Sundascriurus tenuis* (*n* = 2, 4%) and *Rattus rattus* (*n* = 4, 9.3%) were restricted to Saok Waterfalls and Setiu Wetland. Banana and oil palm fruits did not attract any small mammals during the Lasir Waterfall (Hulu Terengganu) survey. All data were interpret into Shannon, Simpson, Margalef, Menhinik and Evenness indices to individually or collectively distinguish small mammal variety in Terengganu State.

**Specifications table**

**Table:** 

Subject area	*Biology*
More specific subject area	Bioscience and Biodiversity
Type of data	Tables
How data was acquired	Cage traps (measuring 28 cm [Length] × 18 cm [Width] × 14 cm [Height]), Vernier caliper (sensitivity 0.1 cm), measuring tape, analytical balance (sensitivity 0.1 kg) and Paleontological Statistics Software Package (PAST) v.3
Data format	Raw and Analyzed
Experimental factors	Cage trap height (between three and five meters) from the ground, spatial placement of cage traps (every ten meters), types of baits (oil palm fruit and banana) used.
Experimental features	Biodiversity indices such as Shannon, Simpson, Margalef, Menhinik and Evenness and, weight to length (W/L) relationships were used to describe the non-volant small mammals from Setiu and Hulu Terengganu districts
Data source location	Setiu District, Terengganu, East Peninsular Malaysia
	Peladang Agro Resort: N 5.5929°; E 102.6797°
	Setiu Wetland Research Centre: N 5.6771°; E 102.7102°
	Hulu Terengganu District, Terengganu, East Peninsular Malaysia
	Lasir Waterfalls: N 4.9655°; E 102.8396°
	Saok Waterfalls: N 5.0832°; E 102.7784°
Data accessibility	All data are available within this article
Related research article	Unpublished data

**Value of the data**

• This data visualizes squirrel, rat and tree shrew abundances in fragmented forest understories along with their morphological descriptions.
• The non-volant small mammal data updates past checklist and compilations (last survey during year 2007), informs about potential agriculture pests (based on bait type) and the variety of non-volant small mammals present in sub-urban and inhabited areas.
• Size and weight data indicate non-volant small mammal growth and food source availability.
• Allows researchers to collaborate, extend their checklist, construct a repository and broaden their statistical analyses.

## 1 Data

This data article is possible after fruit-based (banana and oil palm) baits successfully attracted non-volant small mammals. All trapped small mammal were counted and measured into total length and weight (Table 1) categories. The Evenness, Margalef, Shannon, Menhinik and Simpson diversity indices were calculated to differentiate non-volant small mammals into individual and group diversity values (Table 2). Weight to length (W/L) percentages were used to describe non-volant small mammal growth whereas their statuses in the wild [1] were acquired from IUCN Red List (Table 3). Complete raw data on non-volant small-mammal capture along with additional morphological descriptions are available in a separate list (Table 4).

**Table 1:** Taxonomic classification and abundance of squirrel, rat and shrew abundance discovered from the study sites within districts Setiu and Hulu Terengganu.

Order	Family	Species	Setiu		Hulu Terengganu		*N*	Relative abundance (%)
			A	B	C	D		
Rodentia	Sciuridae	*Callosciurus notatus*	6	10	0	1*	17	39.53
		*Callosciurus nigrovittatus*	1	0	0	0	1	2.33
		*Callosciurus caniceps*	2	0	0	0	2	4.65
		*Sundasciurus tenuis*	0	0	2	0	2	4.65
	Muridae	*Rattus rattus*	0	4	0	0	4	9.30
		*Leopoldamys sabanus*	1	0	0	0	1	2.33
Scandetia	Tupaiidae	*Tupaia glis*	16	0	0	0	16	37.21
Abundance			26	14	2	1	43	100
Species (No.)			5	2	1	1	7	
Field visits (Days)			6	6	6	6	24	
Capture rate (%)			43.3	23.3	3.3	1.7	17.9	

Note: The sites are described as A = Peladang Agro Resort, B = Setiu Wetland Research Station, C = Saok Waterfall and D = Lasir Waterfall. Annotation ‘*N*’ represents number of small mammals and symbol ‘*’ represents number of non-volant small mammals sighted (without contact or handling).

**Table 2:** Calculated diversity indices of non-volant small mammals from the study sites within districts Setiu and Hulu Terengganu.

Indices		Districts		p-Value
		Setiu	Hulu Terengganu	
Diversity Index	Shannon	1.30	0.64	0.67
	Simpson	0.67	0.44	0.67
	Evenness	0.61	0.94	0.96
Richness Index	Menhinick	0.95	1.16	0.48
	Margalef	1.36	0.91	0.77

Note: The *p*-Values were measured using statistical significance up to 95% confidence.

**Table 3:** Identity, statuses in the wild, bait attraction, length to weight percentage and allometric description for non-volant small mammals caught from the study sites within districts Setiu and Hulu Terengganu.

Species	Local name	Status	Bait	W/L (%)	Description
Saok Waterfalls, Hulu Terengganu					
*Sundasciurius tenuis*	Slender squirrel	LC	OPF	27.1 ± 3.1^(*N* = 2)^	NA

Peladang Agro Resort, Setiu					
*Callosciurus caniceps*	Grey-bellied squirrel	LC	OPF	58.7 ± 0.2^(*N* = 2)^	PA
*Callosciurus nigrovittatus*	Black-stripped squirrel	NT	B	22.2 ± 0.0^(*N* = 1)^	NA
*Callosciurus notatus*	Plantain squirrel	LC	OPF	57.7 ± 6.6^(*N* = 3)^	PA
*Callosciurus notatus*	Plantain squirrel	LC	B	55.0 ± 6.4^(*N* = 3)^	PA
*Leopoldamys sabanus*	Long-tailed giant rat	LC	OPF	53.0 ± 0.0^(*N* = 1)^	PA
*Tupaia glis*	Common tree shrew	LC	B	48.5 ± 11.5^(*N* = 15)^	NA

Setiu Wetland Research Station, Setiu					
*Callosciurus notatus*	Plantain squirrel	LC	B	57.6 ± 10.0^(*N* = 8)^	PA
*Rattus rattus*	Black rat	LC	B	37.1 ± 1.3^(*N* = 4)^	NA

Note: Statuses of small mammals follow International Union for Conservation of Nature Red List descriptions whereby LC = Least Concern and NT = Near Threatened. The types of baits used are described as OPF = Oil palm fruit and B = Banana. The Weight-Length ratio represented as W/L are measured using division of weight against total length of animal and measured as percentage (%). The annotations in brackets, ‘*N*’ represents number of animals handled to obtain the desired measurements. Additionally, the Weight to Length (W/L) percentages are described as quartiles represented by <50 % = negative allometric [NA] (Size exceeds body weight), 50 % = symmetric (Body weight increases with size) and >50% = positive allometric [PA] (Body weight exceeds size).

**Table 4:** The unprocessed data of non-volant small mammals caught from study sites within districts Setiu and Hulu Terengganu.

Num.	Year 2017	Day	Time	Species	Bait	Gender	WT (g)	HF (mm)	Ear (mm)	HB (mm)	Tail (mm)	TL (mm)
Saok Waterfall, Hulu Terengganu												
–	02 Aug	1	–	–	–	–	–	–	–	–	–	–
1	04 Aug	2	07.30 a.m.	*Sundasciurius tenuis*	OPF	M	69.1	32	10	126	110	236
2	05 Aug	3	05.30 p.m.	*Sundasciurius tenuis*	OPF	M	59.1	26	12	122	116	238
–	06-07 Aug	4–6	–	–	–	–	–	–	–	–	–	–

Lasir Waterfall, Hulu Terengganu												
–	08-13 Aug	–	–	–	–	–	–	–	–	–	–	–

Peladang Agro Resort, Setiu												
1	14 Aug	1	12.30 p.m.	*Tupaia glis*	B	M	119	34	13	180	155	335
2	14 Aug	1	05.00 p.m.	*Tupaia glis*	B	M	159	35	15	155	172	327
3	15 Aug	2	10.30 a.m.	*Tupaia glis*	B	M	176	35	14	155	160	315
4	15 Aug	2	10.30 a.m.	*Tupaia glis*	B	M	169	33	10	173	165	338
5	15 Aug	2	10.30 a.m.	*Tupaia glis*	B	M	158	34	14	170	165	335
6	15 Aug	2	10.30 a.m.	*Tupaia glis*	B	M	149	40	14	150	170	320
7	16 Aug	3	07.00 a.m	*Callosciurus nigrovittatus*	B	M	256	40	15	179	180	359
8	16 Aug	3	07.00 a.m	*Tupaia glis*	B	M	157	45	15	180	160	340
9	16 Aug	3	07.00 a.m	*Tupaia glis*	B	M	163	37	10	170	160	330
11	17 Aug	4	07.30 a.m.	*Tupaia glis*	B	M	253	38	14	145	150	295
12	17 Aug	4	07.30 a.m.	*Callosciurus notatus*	OPF	M	244	43	13	190	189	379
13	17 Aug	4	07.30 a.m.	*Tupaia glis*	B	M	141	33	11	155	164	319
14	17 Aug	4	06.00 p.m.	*Tupaia glis*	B	M	142	42	14	185	145	330
15	17 Aug	4	06.00 p.m.	*Tupaia glis*	B	M	152	40	15	175	152	327
17	18 Aug	5	06.00 p.m.	*Tupaia glis*	B	M	124	41	12	172	176	348
18	18 Aug	5	06.00 p.m.	*Callosciurus notatus*	OPF	F	219	43	15	208	173	381
19	19 Aug	6	07.00 a.m.	*Callosciurus notatus*	B	M	200	44	14	200	190	390
20	19 Aug	6	05.30 p.m.	*Tupaia glis*	B	M	165	39	13	190	149	339
21	19 Aug	6	05.30 p.m.	*Tupaia glis*	B	M	146	40	14	170	160	330
22	19 Aug	6	05.30 p.m.	*Callosciurus notatus*	B	M	196	43	14	190	192	382
23	19 Aug	6	05.30 p.m.	*Callosciurus notatus*	B	F	224	42	13	175	184	359
24	19 Aug	6	05.30 p.m.	*Callosciurus notatus*	OPF	F	204	44	14	205	194	399
25	19 Aug	6	05.30 p.m.	*Callosciurus caniceps*	B	M	223	45	18	199	180	379
26	19 Aug	6	05.30 p.m.	*Leopoldamys sabanus*	B	M	294	52	25	200	355	555
27	19 Aug	6	05.30 p.m.	*Callosciurus caniceps*	B	M	240	43	15	210	200	410

Setiu Wetland Research Station, Setiu												
1	20 Aug	1	6.0 p.m.	*Callosciurus notatus*	B	M	273	47	19	178	195	373
2	20 Aug	1	6.00 p.m.	*Callosciurus notatus*	B	M	200	45	19	176	193	369
3	20 Aug	1	6.00 p.m.	*Callosciurus notatus*	B	F	198	44	20	175	200	375
4	21 Aug	2	8.00 a.m.	*Callosciurus notatus*	B	M	247	41	20	180	200	380
5	21 Aug	2	6.00 p.m.	*Callosciurus notatus*	B	F	217	42	11	170	174	344
6	22 Aug	3	8.00 a.m.	*Rattus rattus*	B	M	106	36	19	143	135	278
7	22 Aug	3	8.00 a.m.	*Rattus rattus*	B	M	91	30	17	119	120	239
8	22 Aug	3	6.00 p.m.	*Callosciurus notatus*	B	M	169	41	18	180	215	395
9	23 Aug	4	8.00 a.m.	*Rattus rattus*	B	M	102	31	17	120	156	276
10	23 Aug	4	6.00 p.m.	*Callosciurus notatus*	B	M	175	39	14	155	210	365
11	24 Aug	5	8.00 a.m.	*Callosciurus notatus*	B	M	223	41	15	135	225	360
12	24 Aug	5	8.00 a.m.	*Rattus rattus*	B	M	103	30	16	113	179	292
–	25 Aug	6	–	–	–	–	–	–	–	–	–	–

Note: Non-volant small mammal counts are represented by (num.), baits used are denote B = banana and OPF = oil palm fruit and gender are denote with M = male and F = female. Description of measurements are abbreviated as WT = weight, HF = hind foot length, HB = head and body length and TL = total length (from nose tip to end of tail). Measurements are denote g = gram and mm = millimeter.

## 2 Experimental design, materials, and methods

**Fig. 1:**
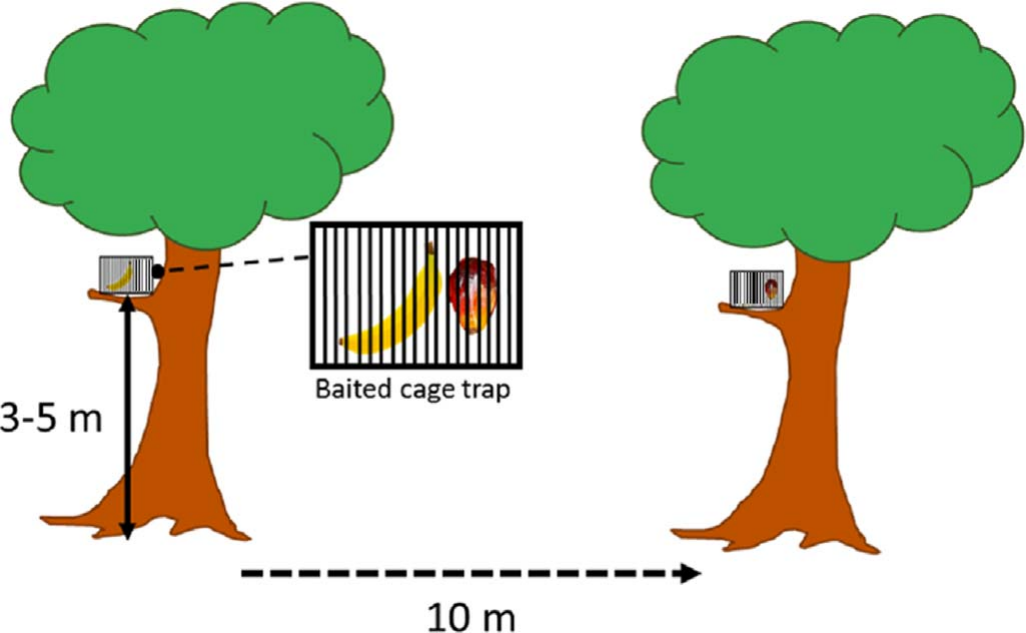
Illustration of cage trap placement on the tree and distances from each trap in the 50 × 50 m transect. The baits used are shown within the figure but each cage has only one type of fruit which differ every 10 m distance and are similar every 20 m distance.

Non-volant small mammal data were gathered from Setiu (Peladang Agro Resort and Setiu Wetland Research Station) and Hulu Terengganu (Lasir and Saok Waterfalls) after twenty-four days by spending six days (five nights) at each site. The experimental design adopted from Lim [2] was used to construct the (50 m × 50 m) 2500 m^2^ transect. Under this setup, fifty cage traps (measuring 28 cm [Length] × 18 cm [Width] × 14 cm [Height]) were baited using either, oil palm fruit or banana pieces (1/4 length). Then, the cage traps were fastened onto tree branches between three (3) and five (5) meters heights. Spatial placement of cage traps were maintained at ten (10) meters apart (Fig. 1). All traps were examined three times daily, between 6.00 a.m. (before sunrise) and 7.00 p.m. (before sunset). The non-volant small mammals were safely secured in cloth bags, examined to distinguish gender and measured for weight, tail length, head-body length, hind foot length, ear-length and total length before their release [3]. Recounting was avoided by excluding non-volant small mammals with trimmed hind leg hairs. Data from the field were transformed into diversity values using Shannon, Simpson, Evenness, Margalef and Menhinik indices available in Paleontological Statistics Software Package (PAST) v.3.
